# Relationship between reflectance and degree of polarization in the VNIR-SWIR: A case study on art paintings with polarimetric reflectance imaging spectroscopy

**DOI:** 10.1371/journal.pone.0303018

**Published:** 2024-05-09

**Authors:** Federico Grillini, Lyes Aksas, Pierre-Jean Lapray, Alban Foulonneau, Jean-Baptiste Thomas, Sony George, Laurent Bigué

**Affiliations:** 1 Department of Computer Science, Norwegian University of Science and Technology, Gjøvik, Norway; 2 IRIMAS, EA7499, Université de Haute-Alsace, Mulhouse, France; 3 ImViA Laboratory—Université de Bourgogne, Dijon, France; USACE ERDC: US Army Engineer Research and Development Center, UNITED STATES

## Abstract

We study the relationship between reflectance and the degree of linear polarization of radiation that bounces off the surface of an unvarnished oil painting. We design a VNIR-SWIR (400 nm to 2500 nm) polarimetric reflectance imaging spectroscopy setup that deploys unpolarized light and allows us to estimate the Stokes vector at the pixel level. We observe a strong negative correlation between the *S*_0_ component of the Stokes vector (which can be used to represent the reflectance) and the degree of linear polarization in the visible interval (average -0.81), while the correlation is weaker and varying in the infrared range (average -0.50 in the NIR range between 780 and 1500 nm, and average -0.87 in the SWIR range between 1500 and 2500 nm). By tackling the problem with multi-resolution image analysis, we observe a dependence of the correlation on the local complexity of the surface. Indeed, we observe a general trend that strengthens the negative correlation for the effect of artificial flattening provoked by low image resolutions.

## 1 Introduction

Specular highlights, shadows, and other atmospheric conditions such as haze are extremely important cues that the human visual system uses to resolve a scene [1–3]. They can provide information regarding the direction of the illumination source, the relative location of objects, and can trigger perceptual effects like color constancy [4, 5]. However, in the framework of image-based material analysis, such *external agents* represent an obstacle in tasks such as characterization and classification [6, 7]. In these instances, the imaging process is affected by a type of noise that results in an ambiguous interpretation of the data, e.g. a naturally light material or a dark material oriented in a way that reflects with specular highlights. In computer vision applications, the task boils down to estimating and discarding the effects of the external agent *E* in the following Eq 1, in which *X* represents the recorded image value and *P* is the *true* property of the material under study.

$$\begin{array}{c} X=f(P,E) \end{array}$$ (1)

In the context of technical imaging of paintings, it is not seldom to encounter challenging glossy targets that display many specular reflections [8, 9]. Usually, the main responsible for the glossy appearance of a painting is the varnish, the outer resin-based layer that is applied with the twofold goal of protection and color enhancement [10]. Specular reflections can also be observed in unvarnished paintings that have been produced with specific pictorial techniques that alter the surface geometry [11]. In *impasto*, for example, large quantities of paint are applied in wide brushstrokes or with a knife, so that the surface is composed of a wide distribution of planar micro facets, from which a resulting specular reflection can be produced. This specular light, therefore, does not come from a single specular plane of incidence, but from a combination of several [12, 13]. Regardless of what causes the specular reflections, the produced pixel values will show saturation, thus preventing any accurate analysis.

Post-processing solutions have been extensively studied to detect and remove the undesired effects introduced by specular reflections [14, 15]. At the same time, it is possible to act at the root of the problem, by carefully designing imaging techniques that can limit the presence of such image flaws [16, 17].

Polarization is a fundamental property of an electromagnetic wave that describes the direction of the electric field oscillation perpendicular to the direction of propagation. The usage of polarization filters is a well-known solution to characterize and classify surfaces [18–22], or to simply reduce the effect of specular reflections in a scene [23, 24]. When carefully designed, a polarization imaging system can allow the detection and removal of such specular components. At the same time, it offers a gateway into looking at the polarization or depolarization effects that are induced by the reflection or transmission of light [25].

When historical artifacts are analyzed by means of imaging, it is crucial to deploy a sustainable technique that does not harm the ongoing preservation and conservation processes. Hyperspectral imaging (HSI), more formally known as imaging spectroscopy, is a non-invasive and non-destructive imaging technique extensively used to study historical artifacts [26].

The combination of polarization and spectral imaging saw the development of Spectral Polarization Imaging (SPI), a technique to capture at the same time polarization and spectral information from a scene. To our knowledge, a compact sensor for the recording of hyperspectral and polarization does not exist yet, so a variety of experimental protocols have been developed for the capturing of such data combinations [27]. SPI is a relatively new field of research with a lot of potential for applications in the context of Cultural Heritage analysis. Most of the literature on SPI has been focusing on the coupling of polarization imaging with multispectral systems, to compactly collect polarization and wide-band spectral data [28–30].

In one of the first studies on SPI, Le Hors *et al.* [31] observed a strong inverse correlation between the degree of linear polarization and the reflectance of diffuse materials such as paints and coatings. It is noteworthy that a similar relationship, termed the *Umov* effect [32] from the Russian astronomer Nikolay Umov who first observed it in 1905, had been observed in the field of astronomy between the albedo of a planet and its degree of linear polarization for large phase angles (intended as the angle between the incident and reflected radiation).

Such correlation is attributed to the individual contributions of surface and volume scattering that take place within the paint layer. The composition of a paint layer can be schematized by a multitude of pigment particles that float in a binding medium (usually a type of oil). In their work, Le Hors *et al.* [31] deployed polarized light and diffusive media, and provided the following explanation of the phenomenon. When polarized incident radiation impinges a surface, a part of it experiences surface scattering and is reflected along the specular direction, maintaining its polarization state. The remaining part of the radiation is either absorbed by the material or experiences volume scattering and is reflected. In the case of absorbance, the final measurement detects a low reflectance with a definite polarization angle (and thus a high degree of linear polarization) for which surface scattering is responsible. In case volume scattering takes place, the outgoing reflected radiation is depolarized but its contribution is greater than the surface-scattered component. Thus, the measurement will detect a high reflectance and a low degree of linear polarization. In the following research, the same team (Le Hors *et al.* [33]) could build a Kubelka-Munk-based model for the description of the depolarization phenomena in paints and other diffuse materials.

The correlation between the degree of linear polarization and reflectance in diffuse materials is an interesting property that can be potentially deployed as a feature for the characterization of materials in conservation science, but it is unknown if it depends on the observed spectral range.

In this article, we propose a paradigm for the acquisition of hyperspectral images in the Visible and Near-Infrared (VNIR) and Short-Wave Infrared (SWIR) in combination with polarization information to investigate in more detail the correlation properties of the interaction between the degree of linear polarization and reflectance. The target used for this study is a mockup oil painting with a rather complex surface topology. The first goal of this paper is to assess the presence of the inverse correlation in the visible range in an imaging context (since the original study by Le Hors *et al.* [31] considered punctual measurements), where the information at the pixel level is affected by several variables. Secondly, we extend the study to the whole available spectrum, and by exploiting the fine spectral sampling we explore how the correlation varies locally through different spectral windows. Finally, we investigate the role of spatial resolution on the computed correlation and try to connect it to the surface properties.

The article is organized as follows. In Section 2, spectral and polarization models and assumptions are provided for a surface exhibiting specular highlights. After deriving predictions on the spectropolarimetric signatures, the acquisition setup and imaging pipeline are presented in Section 3. This will be used as an experiment to confront our predictions with the measurement. We discuss the results in Section 4, before concluding in Section 5.

## 2 Background

### 2.1 Spectral and polarization imaging model

The radiation reflected off a surface depends on the spectral power distribution and the direction of the illumination source, the optical properties of the surface, and the angle between the surface normal and the illumination direction. The Bidirectional Reflectance Distribution Function (BRDF) [34] describes how much radiation is captured by a spectral sensor, factoring in all the previous terms, plus the direction of observation. In Eq 2, *I*_*i*_ and *I*_*r*_ are the incident and observed radiation respectively, while *ω*_*i*_ and *ω*_*r*_ are the illumination and observation direction, respectively.

$$\begin{array}{c} f\left(\omega_{i},\omega_{r},\lambda \right)=\frac{1}{I_{i}\left(\omega_{i},\lambda \right)cos\left(\omega_{i}\right)}\frac{dI_{r}\left(\omega_{r},\lambda \right)}{d\omega_{i}} \end{array}$$ (2)

The BRDF is notably a highly complex model, and cannot be accurately estimated without extensive measurements and expensive setups [35]. However, assuming that a surface is Lambertian can simplify the model by discarding the angular-dependent terms. A Lambertian surface is defined as flat, matte, and diffusive. These three attributes intrinsically contribute to making a Lambertian material isotropic and free from fluorescence phenomena. When the angular terms are discarded, the BRDF coincides with the reflectance of the material under examination:

$$\begin{array}{c} f\left(\lambda \right)=\frac{I_{r}\left(\lambda \right)}{I_{i}\left(\lambda \right)} \end{array}$$ (3)

When specular highlights are observed in an image, it is very clear that the Lambertian assumptions do not hold any longer, and a different model should be used to describe the reflectance behavior.

**Fig 1:**
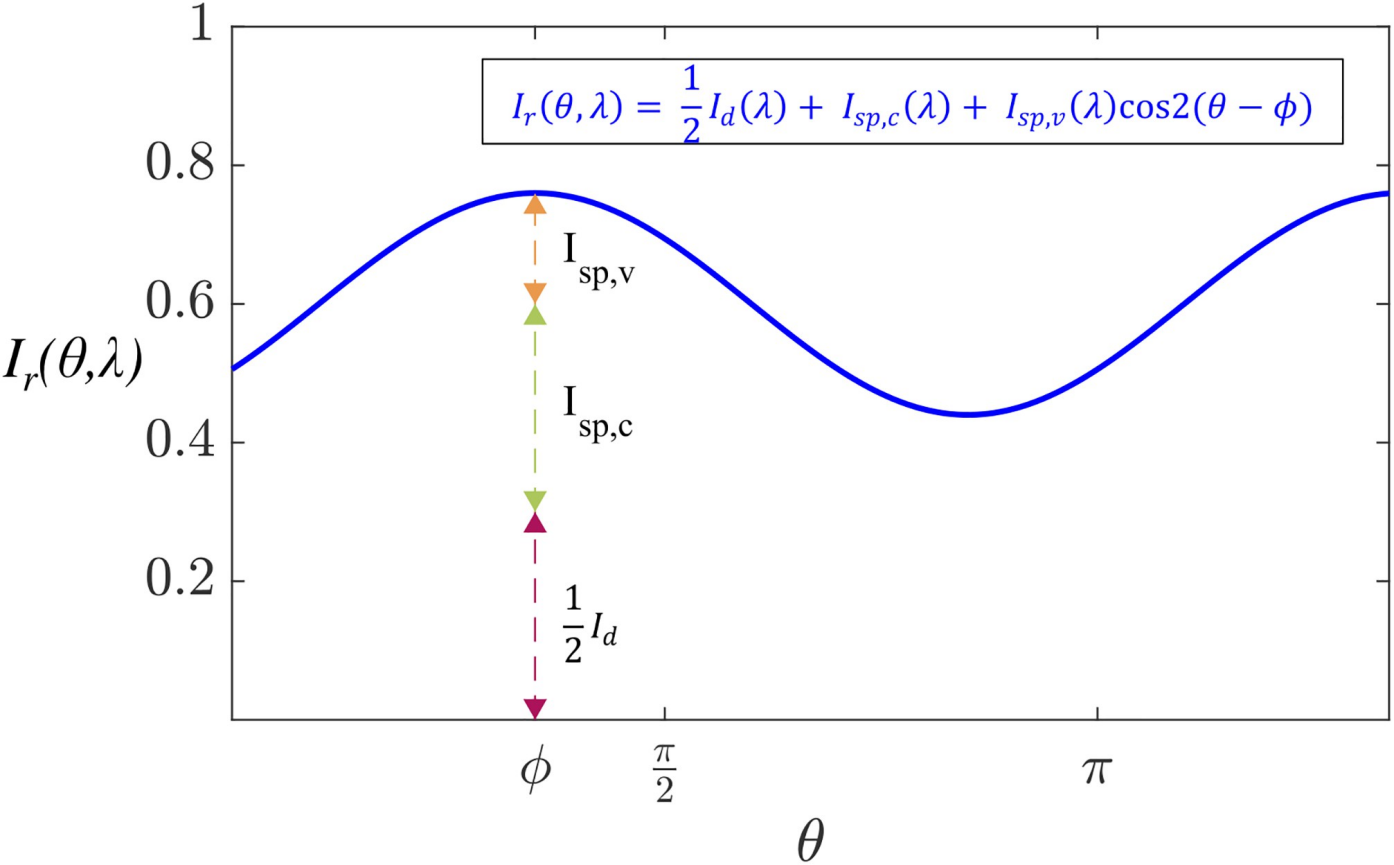
Partially linearly polarized light passing through a linear polarizer after a surface reflection (simulation). The output intensity describes a phase-shifted sinusoid of phase *ϕ*, with constant and variable intensity components.

The dichromatic reflectance model [36] assumes that the reflection of light is composed of a diffuse component (sub-scattering and surface roughness) and a specular component (direct surface reflection). The total intensity *I*_*r*_ after a surface reflection can be modeled by the sum of two intensity components [17], such as:

$$\begin{array}{c} I_{r}\left(\lambda \right)=I_{d}\left(\lambda \right)+I_{s\;p}\left(\lambda \right) \end{array}$$ (4)

where *I*_*d*_ is the diffuse component, assumed to be completely unpolarized, and *I*_*sp*_ is the specular component. The specular reflection has polarization features that depend on the optical properties of the surface interface, i.e. the Fresnel coefficients which are a function of refractive index, wavelength, angle of incidence/reflection, and polarization status of the incident light. In the instance of materials that present multiple sub-surface interactions like paints, it is often assumed that the diffuse component is unpolarized, while the specular component is assumed to be partially polarized. Thus, rotating a linear polarizer with an angle *θ* in front of a camera leads to an intensity measurement such as:

$$\begin{array}{c} I_{r}\left(\theta ,\lambda \right)=\frac{1}{2}I_{d}\left(\lambda \right)+I_{s\;p,c}\left(\lambda \right)+I_{s\;p,v}\left(\lambda \right)\text{cos}\;2(\theta -\phi ) \end{array}$$ (5)

where *I*_*sp*,*c*_ is the constant specular component relative to the angle of the polarization filter, *I*_*sp*,*v*_ is the amplitude of a cosine function term of the variable specular component, and *ϕ* is the angle of linear polarization of light [37]. A visualization of the intensity variation is shown in Fig 1.

Most of the related works measure polarization signals in relatively narrow spectral ranges or with wide spectral bands. This leads to a characterization of polarization that is often limited in terms of spectral analysis. In this work, we capture images of the linear polarization of the reflected light from a surface from 400 nm to 2500 nm sampling at a high spectral resolution.

### 2.2 Stokes vector

The Stokes formalism enables the full description of the polarization state of light with a 4-element vector named the Stokes vector. A conventional Stokes imaging technique combines a camera and a polarizing element to form a Polarization State Analyser (PSA). A linear polarimeter is able to estimate the first three elements of the Stokes vector $S={\left[\begin{array}{cccc} S_{0} & S_{1} & S_{2} & 0 \end{array}\right]}^{t}$, corresponding to the linear polarization state of the incoming light. In particular, *S*_0_ describes the total power of the incident beam, while the *S*_1_ and *S*_2_ components express the difference between intensities measured through orthogonal directions of the polarizer.

The response of a single PSA to a particular input Stokes vector is modeled by the response of a pixel by:

$$\begin{array}{c} \begin{array}{c} I=A\;S\; \end{array} \end{array}$$ (6)

where *I* is the pixel response, *S* is the polarization state of the input light, and $A=\left[\begin{array}{cccc} a_{0} & a_{1} & a_{2} & 0 \end{array}\right]$ is the analyzer vector which embeds the polarizer characteristics of the analyzer components, i.e. transmission, polarizing angle, and extinction ratio coefficient. If ideal transmission and extinction ratio are assumed, the analyzer vector is only a function of the rotation angle, and Eq 6 can be rewritten by:

$$\begin{array}{c} I\left(\theta \right)=\frac{1}{2}[\begin{array}{cccc} 1 & \text{cos}\;2\theta & \text{sin}\;2\theta & 0 \end{array}]S\; \end{array}$$ (7)

We consider a PSA with *M* = 4 discrete polarizer positions, which is enough to estimate *S*. When it is possible, these four angles are selected to be equally spaced in the interval [0, 180]° [38]. In this work, the polarizer is rotated manually, so the angles *θ*_1−4_ are not known. This leads to a vector of intensities **I** defined by:

$$\begin{array}{c} \begin{array}{c} I=[\begin{array}{c} I_{\theta_{1}}\\ I_{\theta_{2}}\\ I_{\theta_{3}}\\ I_{\theta_{4}} \end{array}]=WS=\frac{1}{2}[\begin{array}{cccc} 1 & \text{cos}\left(2\theta_{1}\right) & \text{sin}\left(2\theta_{1}\right) & 0\\ 1 & \text{cos}\left(2\theta_{2}\right) & \text{sin}\left(2\theta_{2}\right) & 0\\ 1 & \text{cos}\left(2\theta_{3}\right) & \text{sin}\left(2\theta_{3}\right) & 0\\ 1 & \text{cos}\left(2\theta_{4}\right) & \text{sin}\left(2\theta_{4}\right) & 0 \end{array}][\begin{array}{c} S_{0}\\ S_{1}\\ S_{2}\\ 0 \end{array}]\; \end{array} \end{array}$$ (8)

in which **W** is the analysis matrix that combines the four analyzer vectors *A* and *θ*_1−4_ are the polarizer angles of the four PSA configurations. The four angles are estimated through a calibration procedure (described in Section 3.3).

Once **W** is estimated, the Stokes vector $\hat{S}={\left[\begin{array}{cccc} {\hat{S}}_{0} & {\hat{S}}_{1} & {\hat{S}}_{2} & 0 \end{array}\right]}^{t}$ can be computed from *I* for each pixel in the image, such as:

$$\begin{array}{c} \hat{S}={\hat{W}}^{+}I. \end{array}$$ (9)

in which ${\hat{w}}^{+}$ is the pseudo-inverted PSA matrix estimation. For a polarimeter that has *K* spectral bands, the Stokes vector is estimated by spectral band *k* as follows:

$$\begin{array}{c} {\hat{S}}_{k}={\hat{W}}^{+}I_{k}. \end{array}$$ (10)

A more intuitive set of polarimetric parameters can be computed from Stokes vectors, which are the degree of linear polarization *ρ*_*k*_ and the angle of linear polarization *ϕ*_*k*_:

$$\begin{array}{cccc} \rho_{k} & =\frac{\sqrt{S_{1,k}^{2}+S_{2,k}^{2}}}{S_{0,k}} & \phi_{k} & =\frac{1}{2}\text{arctan}(\frac{S_{2,k}}{S_{1,k}}) \end{array}$$ (11)

The manually rotated PSA has a transmission axis *θ* (with respect to a reference angle). The transmitted light is thus linearly polarized along this axis but with an intensity attenuated by a specific amount, modeled by a cosine law [19]: *I*(*θ*) = *I*_0_ cos^2^ *θ*.

## 3 Materials and methods

### 3.1 Experimental set-up

The hyperspectral cameras deployed in this study are of the type *push-broom*. They are sensitive to the VNIR (Hyspex VNIR1800, Norsk Elektro Optikk) and SWIR (Hyspex SWIR384, Norsk Elektro Optikk). The VNIR image sensor, manufactured in Silicon (CMOS), captures radiation from 400 nm to 1000 nm with 186 spectral channels and with 1800 pixels on the acquisition line, whereas the SWIR sensor (Mercury-Cadmium-Telluride) is sensitive in the interval 950 nm to 2500 nm with 288 spectral channels and 384 pixels on the acquisition line.

**Fig 2:**
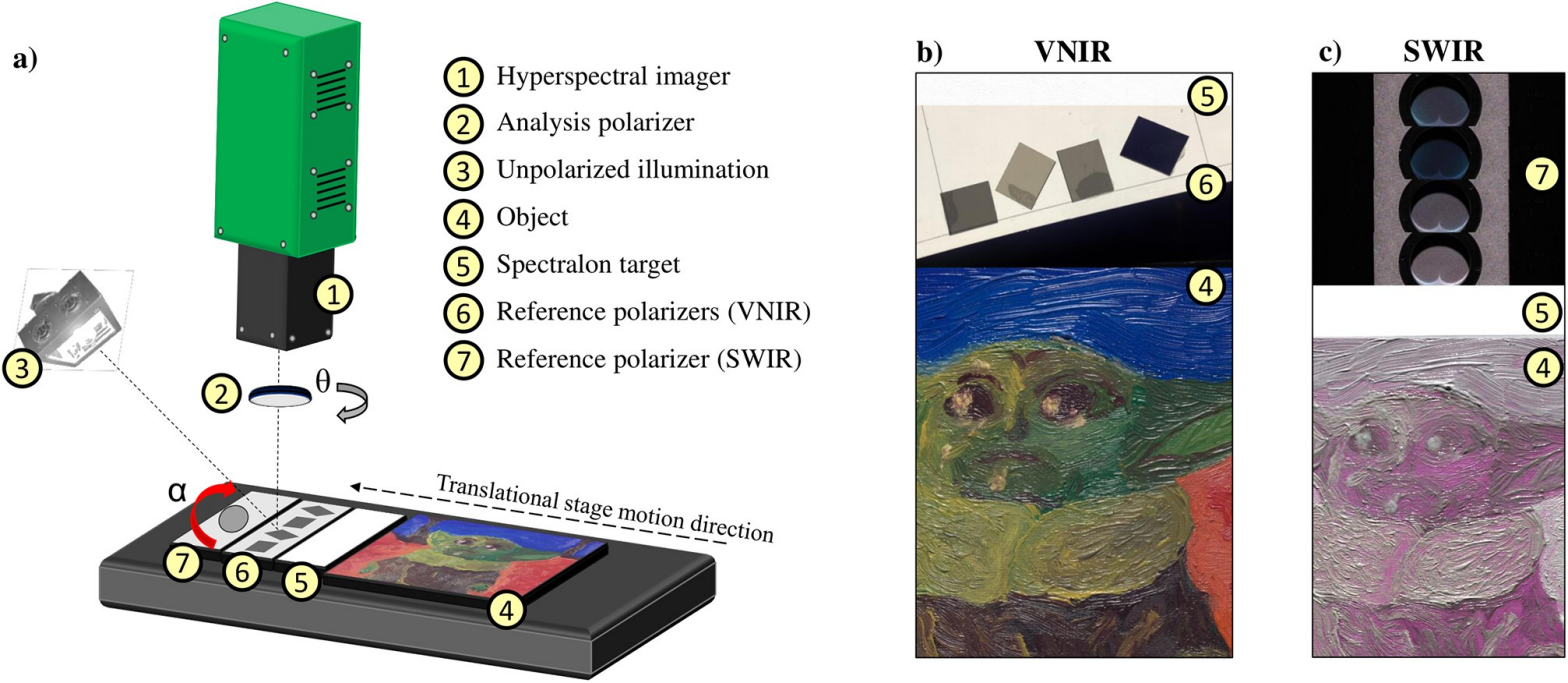
Imaging setup. **a)** Schematized experimental setup with a legend for the corresponding elements. The illumination source and the elements that form the scene must be kept in place when the imager is changed (from VNIR to SWIR for example), in order to keep the BRDF as close as possible between the two hyperspectral modalities. **b)** Color rendering of an example scene in VNIR. **c)** False color infrared of an example scene in SWIR in which the images of the reference polarizers have been stacked in post-processing.

Hyperspectral images in VNIR and SWIR were captured with different but highly similar setups, schematized in a single one in Fig 2a. Due to space limitations, it was not possible to deploy both cameras simultaneously, so we opted to acquire VNIR and SWIR images sequentially.

**Fig 3:**
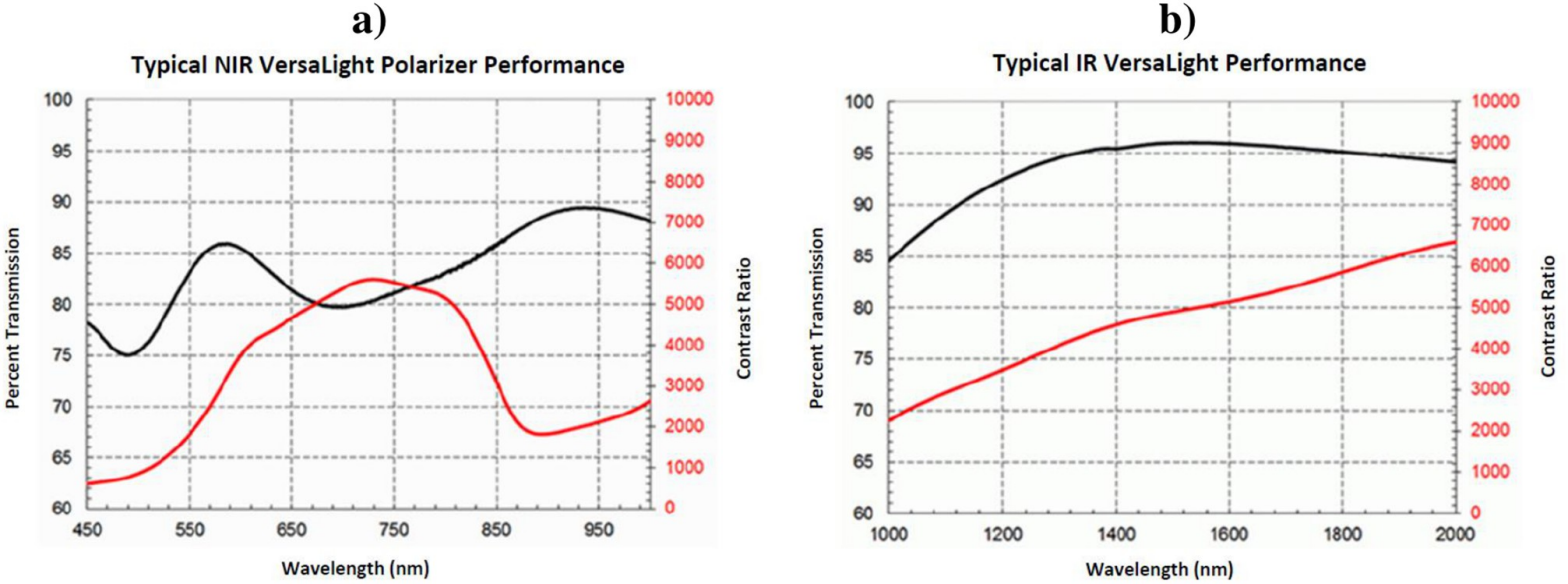
Polarization characteristics of the analysis polarizers placed in front of the hyperspectral imagers. **a)** VNIR analysis polarizer. **b)** SWIR analysis polarizer. The figures are displayed as reported in [39].

An analysis polarizer (also called analyzer, represented by *θ*) is placed before the camera objective and can be rotated manually at each new capture. The VNIR polarizer is a 1” Meadowlark NIR Versalight wide-grid polarizer (VLR-100-NIR), while the SWIR camera is coupled with a 1” Meadowlark IR Versalight wide-grid polarizer (VLR-100-IR) [39]. These linear polarizers are built with aluminum nanowires and their contrast ratio, reported in Fig 3, ensures an efficient usage in a broadband spectral range. Indeed the contrast ratio is constantly higher than 500 for the VNIR polarizer, and constantly above 2000 for the SWIR polarizer, which is an indication of reliability.

Previous research [40] aimed at characterizing Hyspex VNIR1600 and Hyspex SWIR320m-e (two earlier models of the ones used here) observed a maximum 10% polarization sensitivity in both cameras in the first spectral bands of the individual sensors, with the average sensitivity stabilizing at around 5%. We can assume that this observation is still in place for the sensors deployed in this work, but the reported polarization sensitivity does not impact the measurements significantly, since the polarizers added in front of the cameras are highly efficient compared to the inherent sensitivity.

In this push-broom configuration, the translational stage slides across the field of view of the camera at a speed synchronized with the framerate of the camera, while a halogen light shines a constant unpolarized light flux exactly on the acquisition line of the camera.

A substantial difference with the study by Le Hors *et al.* [31] is the deployment in our setup of unpolarized light. This implies that it is not possible to strictly talk about depolarization effects and that any polarization that will be recorded by the imagers is induced upon reflection.

The following elements are placed on the translational stage: the object to be captured, a Spectralon (LabSphere) target for spectral calibration, and a reference polarizer. In the case of the VNIR capture, four cutouts of the same linear polarizer sheet (model XP40HT-40, Edmund Optics) are placed at known *α*_1−4_ angles separated by 45° from each other. Due to the unavailability of a sheet that could polarize light in the SWIR range, a single polarizer working in the same range (model LPNIRC100-MP2, Thorlabs) was placed on a motorized rotational stage. The spectral ranges in which the deployed reference polarizers are effective are between 400 nm and 700 nm for XP40HT-40, and between 1100 nm and 1800 nm for LPNIRC100-MP2. This means that the spectral ranges of the cameras are much wider, and therefore only those spectral bands that present an acceptable attenuation will be considered for the polarimetric calibration step.

The target object selected for this experiment is a mockup oil painting that presents quite a lot of specular reflections due to its topology. The pre-primed cotton canvas received two additional priming layers of gesso, while seven pigments were bound with linseed oil and applied either in their pure state or mixed combinations. The painting is unvarnished, thus the observed specular reflections are caused by wide brushstrokes that were applied in an attempt of replicating the *impasto* technique. At the time of the imaging campaign, the mockup painting had aged for two and a half years in a dry and dark environment (not controlled in temperature or relative humidity).

**Fig 4:**
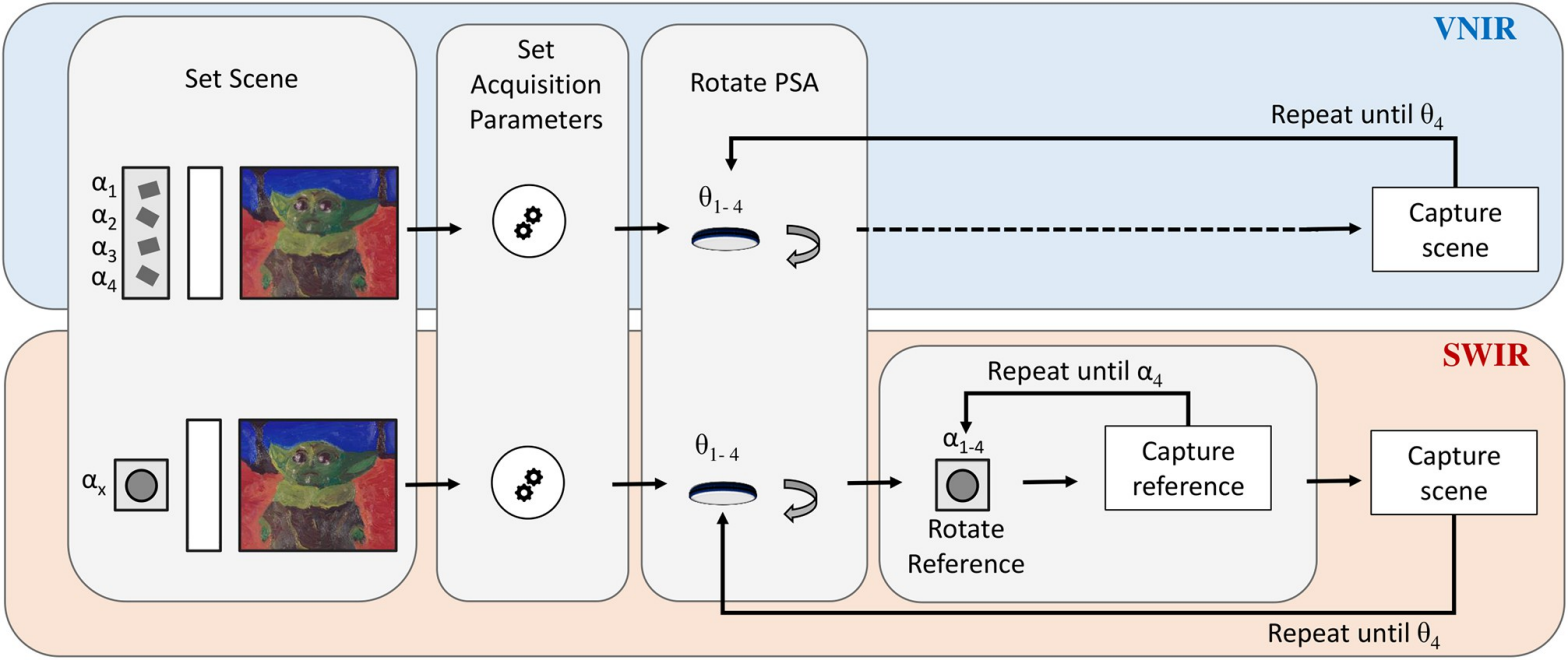
Schematized acquisition paradigm for combined VNIR-SWIR hyperspectral polarization imaging.

To summarize, both VNIR and SWIR capture processes require the sequential acquisition of four images with a fixed integration time, each corresponding to a new rotation of the analysis polarizer by 45°. The only difference between VNIR and SWIR resides in the fact that at each manual rotation of the SWIR analyzer, four separate acquisitions of the reference polarizer at angles *α*_1−4_ are needed in order to replicate the situation of the four reference polarizers lined up in the VNIR setup. The procedure is schematized in Fig 4 while an example of the captured VNIR and SWIR scenes is reported for *θ*_2_ in Fig 2b and 2c. Here, the sequentially captured SWIR images of the reference polarizer are stacked on the top of Fig 2c.

### 3.2 Spectral data preprocessing

The hyperspectral cameras readily provide image cubes of raw data that need to undergo a series of correction and calibration steps before obtaining absolute reflectance data. A first geometric correction [41] is performed to account for distortions in the across-track direction that arise from differences in viewing angles for the pixels on the acquisition line. Then, a radiometric correction is performed to transform the data from raw to relative radiance, discarding the effects of constants, and user and camera-dependent parameters.

Flat-fielding correction deploys the reflectance target placed in the scene, but it can be performed also with a non-standardized target, as long as the surface is uniform and diffusive. The light field recorded by the reflectance target can be non-homogeneous due to the manual positioning of the light source, so this step accounts for this type of distortion, transforming the radiance data into relative reflectance. Absolute reflectance data is then obtained by using the provided reflectance values of the standardized Spectralon target. Eq 12 summarizes the procedure:

$$\begin{array}{c} \beta \left(x,\lambda \right)=\frac{L(x,\lambda )}{\eta (x,\lambda )}\cdot \frac{\chi (\lambda )}{\mu (\lambda )} \end{array}$$ (12)

Here, a pixel with radiance value *L*(*x*, λ) is divided by the corresponding radiance value on the flat-fielding target *η*(*x*, λ) and then multiplied by the ratio between the reference reflectance of the Spectralon target *χ*(λ) and the relative reflectance (flat-fielded) extracted from the Spectralon in the scene *μ*(λ).

In the capturing sequence, it was decided to first acquire all images in the VNIR range, and subsequently, all the images in the SWIR range, while the painting mockup was kept in place. This allows us to assume that all images within the same spectral range are co-registered, while there exists a unique geometric transform (homography) that connects the VNIR set to the SWIR set. The homography matrix is learned with a first step of SIFT feature matching [42], followed by a refining step using the methodology proposed in [43]. It is important to point out that only the mockup painting portion of the scene is registered, while the Spectralon tile and the polarization reference filters are cropped out. At this stage, the images are registered at SWIR resolution, therefore downgrading the quality of the VNIR set. Later in the article, we will point out the analysis sections that consider the VNIR and SWIR ranges jointly, and those steps that consider them independently, therefore using the VNIR set at its full spatial resolution.

### 3.3 Polarimetric calibration

The polarimetric calibration is the estimation of the four analysis angles *θ*_1−4_ for each of the two cameras. It is performed in three steps: 1- measure the relative angles of the reference polarizing filters *α*_1−4_, 2- take the intensity values corresponding to the reference polarizers in the scene, and 3- fit a cosine function on reflectance data to find *θ*_1−4_.

The VNIR reference polarizers are lined up on a supportive sheet (see Fig 2) and their relative angles *α*_1−4_ are measured with a high-performance polarization filter array camera from Lucid Vision Labs, featuring an on-chip SONY IMX250 MYR [44] sensor. Due to the usage of an electronically controlled rotational stage, the reference angles corresponding to the SWIR captures are directly known as input.

The following values are the orientations of the fixed reference polarizers:

$\alpha_{V\;N\;I\;R}=\left[\begin{array}{cccc} 0{}^{\circ} & 48.60{}^{\circ} & 92.75{}^{\circ} & 141.40{}^{\circ} \end{array}\right]$ (from left to right in Fig 2b)

$\alpha_{S\;W\;I\;R}=\left[\begin{array}{cccc} 10{}^{\circ} & 55{}^{\circ} & 100{}^{\circ} & 145{}^{\circ} \end{array}\right]$ (from top to bottom in Fig 2c)

**Fig 5:**
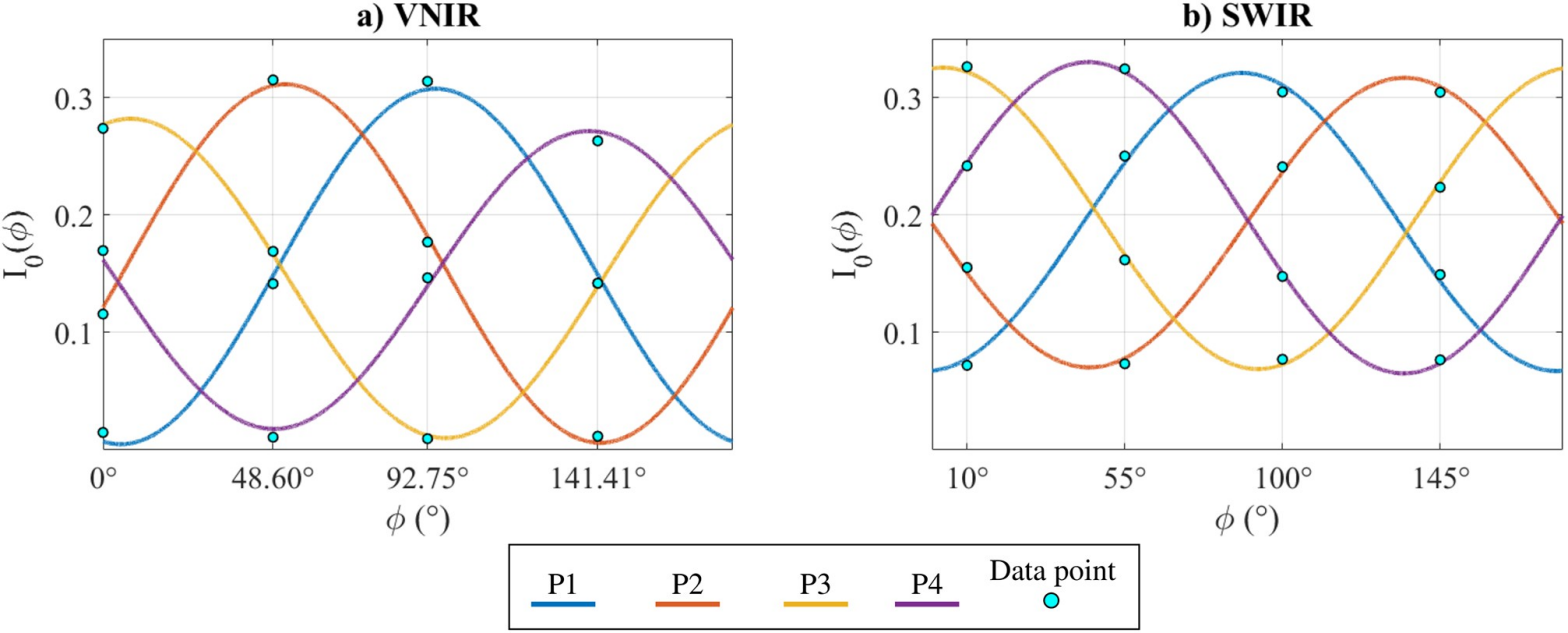
Example of fitting of a cosine law from intensity measurements of reference polarizers, for a single spectral band. This allows to estimate the angles of analysis *θ*_*VNIR*_ and *θ*_*SWIR*_ from the phases. **a)** band corresponding to 564 nm. **b)** band corresponding to 1497 nm.

The cosine law is then fitted with the least square method on the intensity values extracted from the reference polarizers. Then, the analyzer angles are estimated by finding the phase of each fitted curve. Fig 5 displays an example of the fitting at 564 nm for the VNIR camera and at 1497 nm for the SWIR camera.

**Fig 6:**
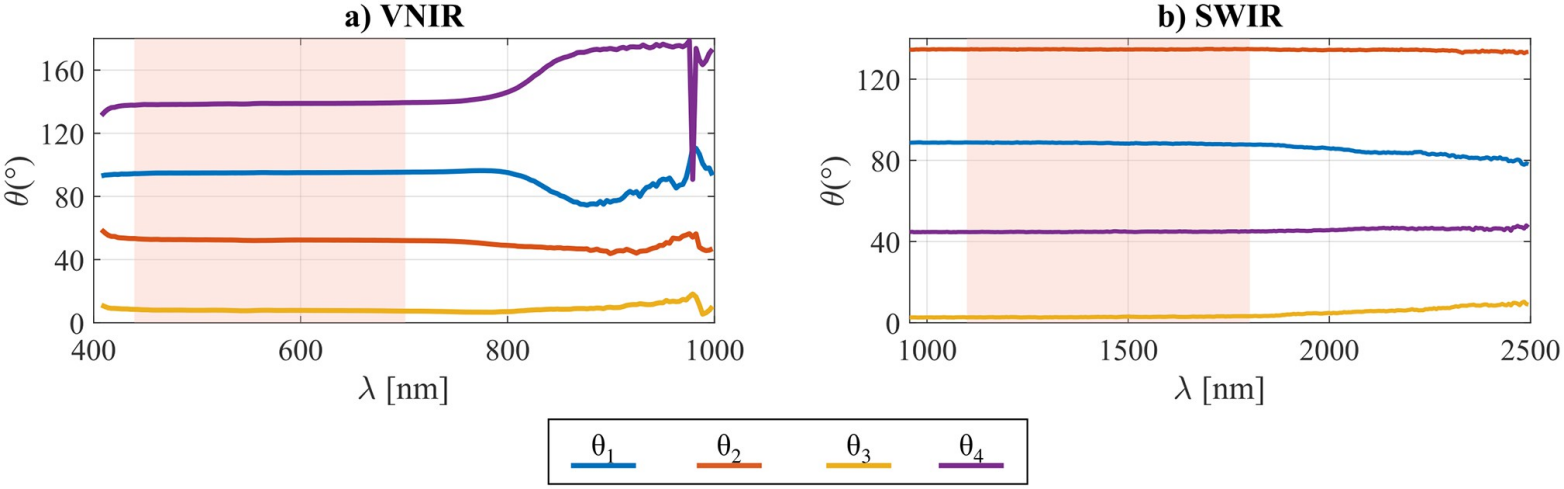
Results of estimated analysis angles *θ*_1−4_ at each spectral band. The shaded areas between 440 nm and 700 nm (**a)** VNIR) and between 1100 nm and 1800 nm (**b)** SWIR) represent the spectral range in which the reference polarizers are known to have a high extinction ratio, and consequently, the average *θ*_1−4_ values are computed from those spectral bands.

The analysis angles *θ*_*VNIR*_ and *θ*_*SWIR*_ are estimated in correspondence with the phases of the curves, and their behavior as a function of wavelength is reported in Fig 6.

The reliability of the estimation depends on the extinction ratios of the deployed reference polarizers, which are known to be effective from 400 to 700 nm for the VNIR acquisition, and from 1100 to 1800 nm for the SWIR acquisition. Since the first spectral bands of the VNIR camera are more affected by noise, the considered spectral range is limited to 440 to 700 nm. The following *θ*_*VNIR*_ and *θ*_*SWIR*_ are then computed as the average in the high-extinction ratio spectral ranges (red shaded areas in Fig 6):

$\theta_{V\;N\;I\;R}=\left[\begin{array}{cccc} 94.94{}^{\circ} & 51.98{}^{\circ} & 7.78{}^{\circ} & 139.22{}^{\circ} \end{array}\right]$

$\theta_{S\;W\;I\;R}=\left[\begin{array}{cccc} 88.26{}^{\circ} & 134.28{}^{\circ} & 2.94{}^{\circ} & 44.88{}^{\circ} \end{array}\right]$

The consistent results obtained by wavelength in the polarimetric calibration serve as a health check to demonstrate that the procedure, although including elements of human interaction (manual rotation of analysis polarizer), is accurate enough to retrieve the Stokes vectors pixel-wise and spectrally. Nonetheless, the setup would benefit from the implementation of a motorized system for the rotation of the analysis polarizer.

We then compute the analysis matrix **W** from Eq 6:

$$\begin{array}{cccc} W_{V\;N\;I\;R} & =[\begin{array}{cccc} 0.5 & -0.49 & -0.08 & 0\\ 0.5 & -0.12 & 0.48 & 0\\ 0.5 & 0.48 & 0.13 & 0\\ 0.5 & 0.07 & -0.49 & 0 \end{array}] & \;\;\;\;\;W_{S\;W\;I\;R} & =[\begin{array}{cccc} 0.5 & -0.49 & 0.03 & 0\\ 0.5 & -0.01 & -0.49 & 0\\ 0.5 & 0.49 & 0.05 & 0\\ 0.5 & 0.00 & 0.5 & 0 \end{array}] \end{array}$$

The resulting **W**_**VNIR**_ and **W**_**SWIR**_ values are then used, according to Eq 10, to retrieve the Stokes vector images in a band-wise fashion. The respective condition numbers for **W**_**VNIR**_ and **W**_**SWIR**_ (excluding the fourth columns full of zeros) are 1.438 and 1.446, close to the ideal value of $\sqrt{2}$. Hence, our measurement process will provide polarimetric estimates with reduced noise [38].

### 3.4 Spectro-polarimetric splicing

When spectral information is captured with two different sensors in adjacent or overlapping spectral ranges, it is common to observe discrepancies in their responses. This is usually due to a series of factors that include different spectral bandwidths, low signal-to-noise ratio, and misalignments in the imaging setup that cause the BRDF to slightly vary. Spectral splicing is a correction that smoothly connects two spectra affected by *spectral jumps* [45], and can be extended to connect VNIR and SWIR hyperspectral images [46], like in our instance.

However, the VNIR and SWIR hyperspectral images cannot be readily connected as they are captured, since the polarization information has been collected at different angles of the PSA. We then propose to apply the splicing correction on the two independent Stokes multi-band images, in order to obtain a full range version of *S*_0_ and *ρ* that is continuous in the range between 400 nm and 2500 nm.

### 3.5 Correlation between reflectance and degree of linear polarization

Le Hors *et al.* [31] observed a strong negative correlation between the proportion of reflected radiation and the degree of linear polarization of diffusive media such as paints in the visible spectral range, between 400 nm and 780 nm. One of the goals of this article is to corroborate this observation also in an imaging framework at the pixel level and verify its validity in the spectral range spanning from 400 nm to 2500 nm. As the work by Le Hors *et al.* sets a precedent, we opted to study the correlation between reflectance and degree of linear polarization, considering the ${\hat{S}}_{1}$ and ${\hat{S}}_{2}$ components of the Stokes vector as intermediate products of challenging interpretability if taken individually. Furthermore, the angle of linear polarization is not considered in the present study, but it can be included in a future phenomenological investigation.

After having computed the Stokes vectors pixel-wise, the first element ${\hat{S}}_{0}$ is selected to represent the spectral reflectance of the pixel, as per its definition, it is proportional to the pixel reflectance. We term ${\hat{S}}_{0}$ as *pseudo-reflectance*, to distinguish it from the scene reflectance that can be computed in polarimetric systems [47]. From the Stokes vector, the degree of linear polarization *ρ* is computed with Eq 11.

The global correlation coefficient can be computed as:

$$\begin{array}{c} R_{g}=\frac{\sum_{k=1}^{K}\left(\rho_{k}-\bar{\rho }\right)\left(S_{0k}-{\bar{S}}_{0}\right)}{\sqrt{\sum_{k=1}^{K}{\left(\rho_{k}-\bar{\rho }\right)}^{2}}\sqrt{\sum_{k=1}^{K}{\left(S_{0k}-{\bar{S}}_{0}\right)}^{2}}} \end{array}$$ (13)

By computing the correlation at the pixel level, it is possible to obtain a spatial map of correlation. The polarization of reflected light has a strong dependence on the surface topology. Since the mockup painting presents a distinct roughness, the information regarding the polarization properties of a material can be very unstable in a local neighborhood. We then propose to investigate the behavior of the correlation when subjected to a multi-resolution approach. By doing so, it is possible to pull together groups of neighboring pixels, so that the surface is artificially flattened, in the case where multiple surface normals coexist, or uniformed toward a dominant normal direction.

Typically, multi-resolution approaches make use of techniques such as Gaussian blur or Laplacian Pyramids, but these methods do not consider the spatial structures and patterns that exist in a scene, thus pooling neighboring pixels together indiscriminately. We propose to segment the scene using the SLIC superpixel technique [48]. In this way, similar neighboring pixels are pushed together while the spatial structures of the image are still recognizable. We argue that by doing so, all pixels within a superpixel are constituted of the same material.

Then, for each superpixel, we compute the new corresponding Stokes vector, the degree of linear polarization, and the correlation.

Another property of correlation that we want to investigate is its dependence on the considered spectral range. This can be studied by computing a local measure of correlation [49] within a predefined spectral window of width *w* as:

$$\begin{array}{c} R_{l}\left(k\right)=\frac{\sum_{i=k-w/2}^{k+w/2}\left(\rho_{i}-\bar{\rho }\right)\left(S_{0i}-{\bar{S}}_{0}\right)}{\sqrt{\sum_{i=k-w/2}^{k+w/2}{\left(\rho_{i}-\bar{\rho }\right)}^{2}}\sqrt{\sum_{i=k-w/2}^{k+w/2}{\left(S_{0i}-{\bar{S}}_{0}\right)}^{2}}} \end{array}$$ (14)

## 4 Results and discussion

### 4.1 Decision on the number of superpixels

The SLIC algorithm allows to over-segment an image into perceptually similar neighborhoods of irregular shapes. The first question that we need to answer is which image is taken as a reference for the computation of the superpixel masks. Since the SLIC algorithm was originally designed to work on color images, it is more appropriate to work on an RGB representation of the scene. The four VNIR images acquired at the different analysis angles *θ*_1−4_ record specular reflections with slightly different intensities and patterns, so generating superpixels from an individual image captured at *θ*_*i*_ might not be able to generalize for the remaining three instances. For this reason, it was decided to generate a new RGB image from a *θ*-independent version of the scene, represented by the *S*_0_ component of the Stokes vector computed from the RGB intensity values.

**Fig 7:**
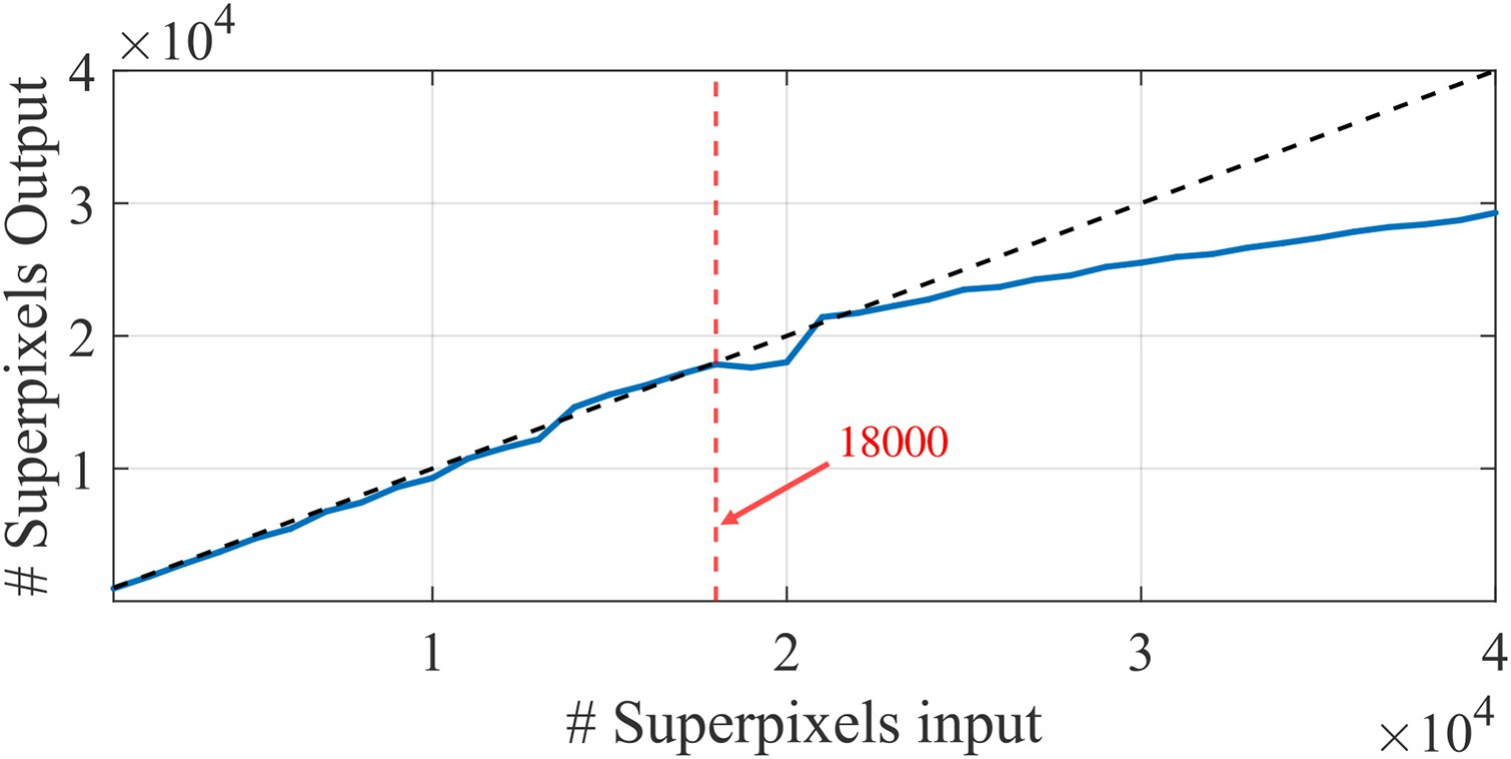
Decision on the maximum number of superpixels. The breaking point of linearity is observed around the input of 18000 superpixels.

The subsequent questions concern the number of multi-resolution steps and the number of superpixels that each step should have. The only inputs required by the SLIC algorithm are an image and the approximate number of superpixels. The algorithm will then output a number of superpixel masks close but not equal to the one provided as input. To find out the maximum number of superpixels, we graphically look for the breaking point of the linear relationship between input/output. This is illustrated in Fig 7.

After having selected 18000 as the maximum number, we progressively decrease by a factor of 1.5, thus obtaining the following list of superpixel numbers: 18000, 12000, 8000, 5333, 3555, 2370, 1580, 1053, 702, 468, 312, 208, 139, 93, 62, 41, 27, 18. It is important to point out that for a given application the number of deployed superpixels is highly dependent on the scene content. Moreover, the final decision regarding what edges to preserve is also affected by subjectivity and the purpose of use.

**Fig 8:**
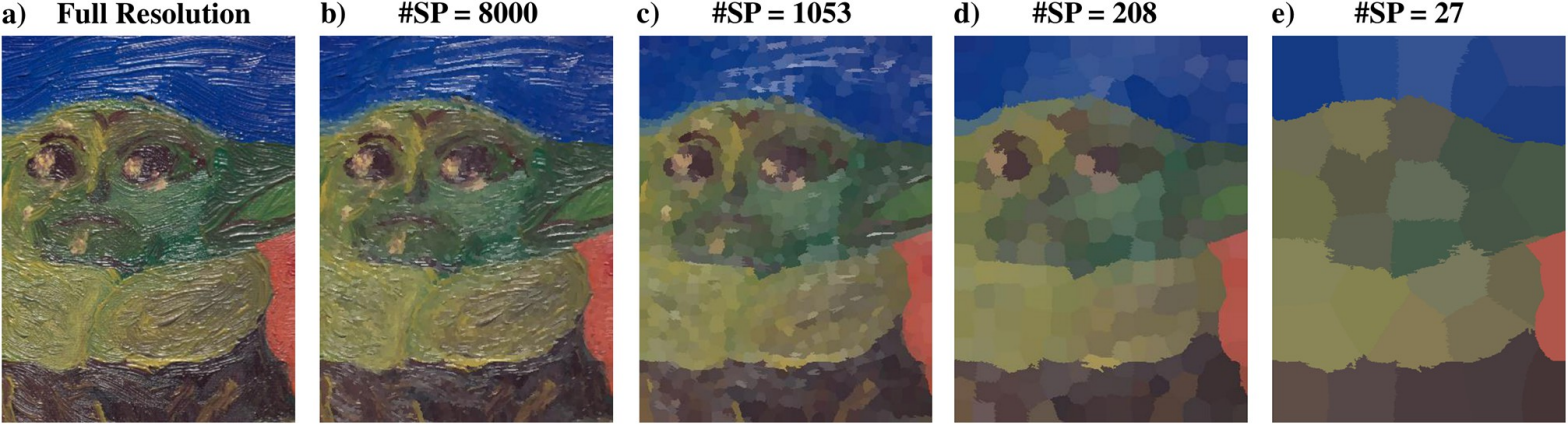
Examples of over-segmentation with SLIC superpixels on the image obtained from the *S*_0_ element of the Stokes vector. The most relevant edges of the scene are preserved even with a very small number of superpixels.

Fig 8 displays some selected results of the over-segmentation by reporting the average color values within the superpixel masks.

### 4.2 Global correlation: Scale and wavelength dependence

Investigating the properties of an observed correlation can provide further analysis into discovering the underlying relationship between two quantities. In this instance, it was previously observed that the reflectance and the linear degree of linear polarization are strongly correlated (negatively) in the visible range.

**Fig 9:**
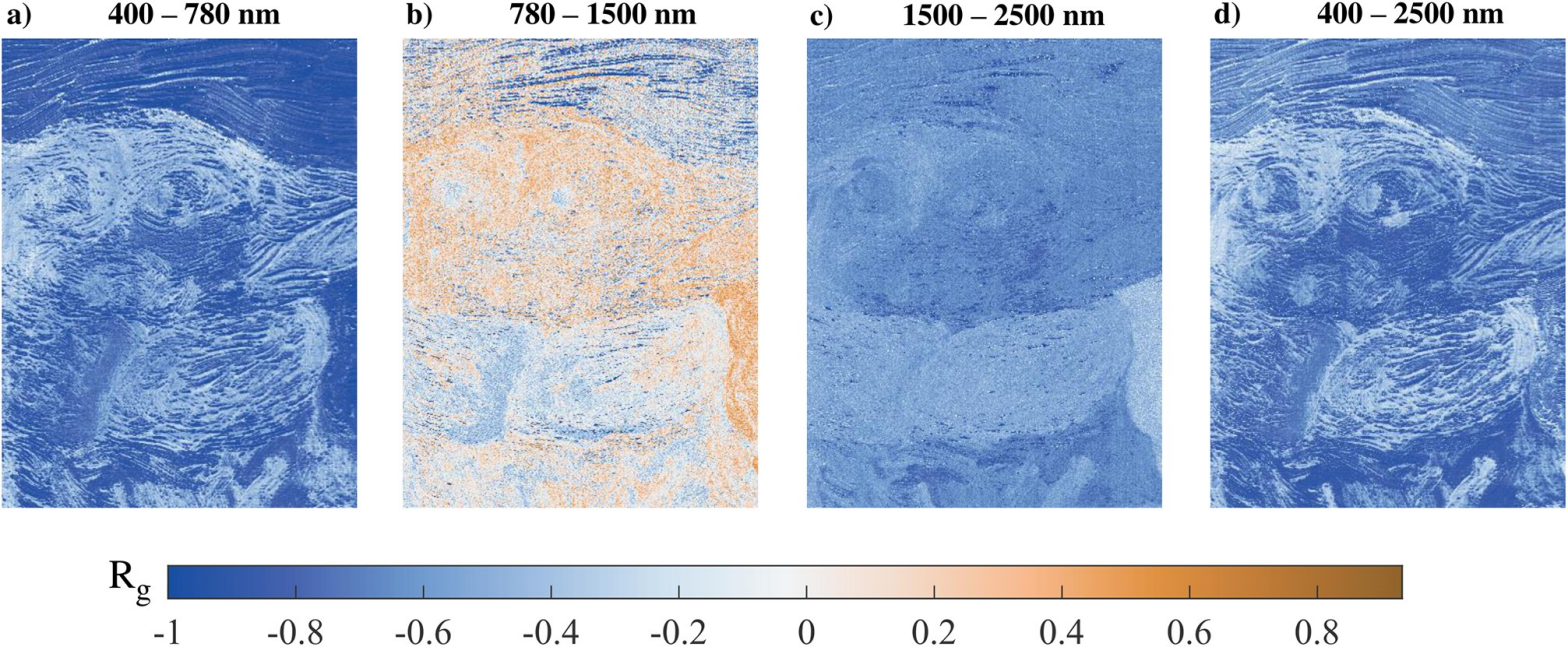
Global correlation (*R*_*g*_) maps in different spectral ranges. Besides the evident variation in correlation magnitude between the different spectral ranges, it is also possible to observe the change in the distribution of highly correlated/decorrelated areas.

The first approach that we propose, is to verify this hypothesis at the pixel level on the scene constituted by the mockup painting, for four different spectral ranges: 400-780 nm, 780-1500 nm, 1500-2500 nm, and the full range 400-2500 nm. Fig 9 illustrates this. The original assumption is promptly verified in the visible range (400—780 nm, Fig 9a), but something interesting happens as we shift towards longer wavelengths. The first part of the infrared range (780—1500 nm, Fig 9b) shows a marked decorrelation between *S*_0_ and *ρ*, while a more strong negative correlation comes back for a deeper interval in the infrared (1500—2500 nm, Fig 9c). The global correlation in the full range (Fig 9d) seems to provide a sort of average, although correlation is a non-linear quantity and this can sound misleading.

To understand the fluctuation of correlation when moving towards longer wavelengths, it is perhaps necessary to refer to the interaction of infrared radiation with the painting layer. Often, paintings are investigated with infrared radiation to reveal underdrawings and *pentimenti* [50] underneath the paint layer, exploiting the transmission properties of pigments in this spectral range. In the range from 780 nm to 1500 nm, the radiation gets trapped in a series of volume scatterings and upon exiting the material its degree of linear polarization is very small. An almost zero and constant degree of linear polarization then produces the decorrelation values observed in Fig 9b.

We argue that the observed strengthening of negative correlation in the range between 1500 nm and 2500 nm is counter-intuitive. Longer wavelengths should penetrate deeper into the material and generate more volume scattering, thus displaying an even lower degree of linear polarization. Polarization of the radiation could be observed if the infrared light interacted with the underneath preparatory layer coated with gesso. However, we exclude this possibility as we know by construction that the painting layer is thick enough to absorb all radiation in the considered spectral range.

It is interesting to observe how some areas change their behavior when switching the considered spectral range. Take for instance the area on the right side that is red in Fig 8. This area shows a strong negative correlation in the visible range, then a relatively strong positive correlation in the near-infrared, and finally a decorrelation in the deeper infrared range. This peculiar behavior could be ascribed to a particular pigment, in this instance Vermilion, but further analyses are required.

**Fig 10:**
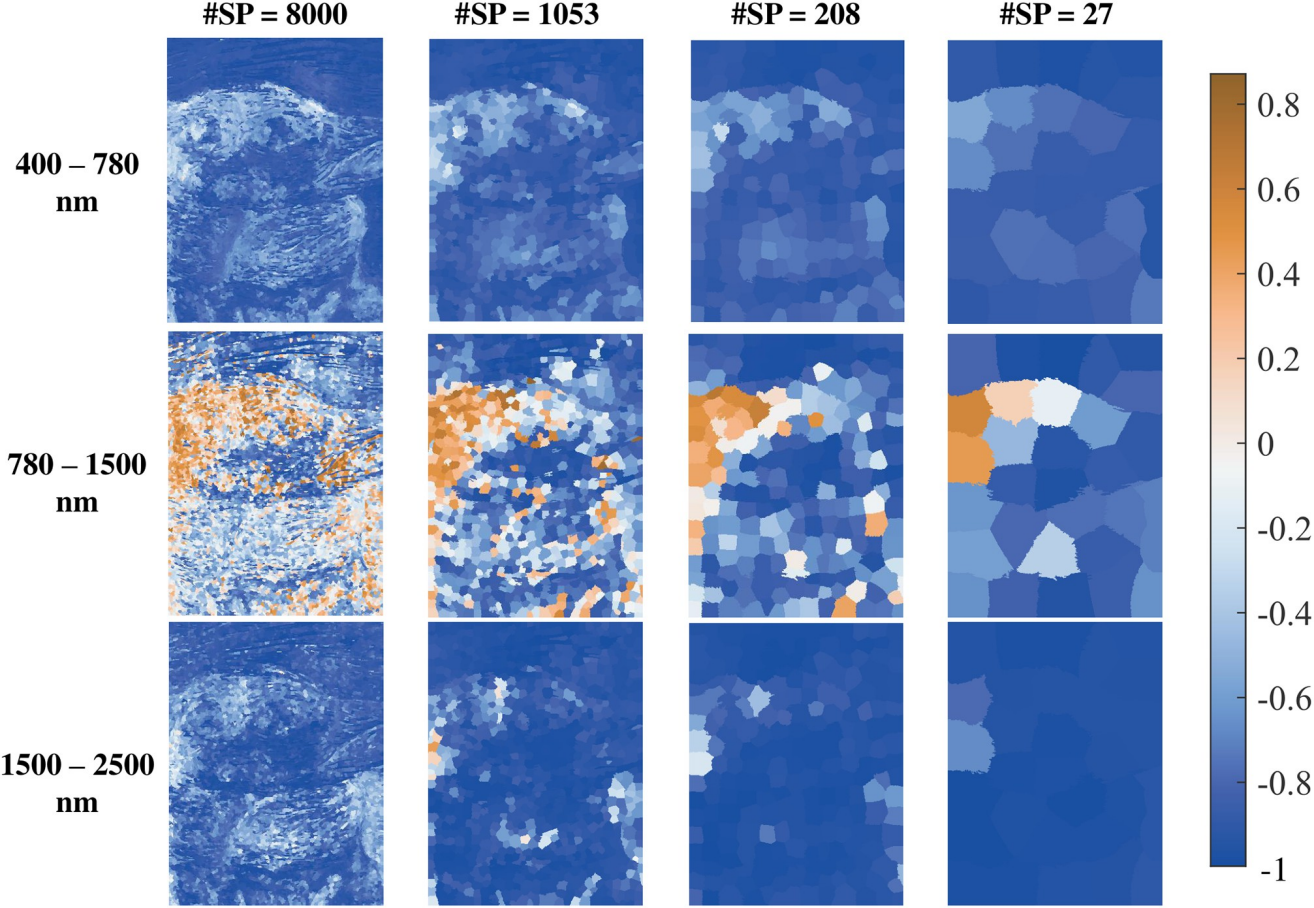
Global correlation (*R*_*g*_) maps in different spectral ranges and for different numbers of superpixels.

In Fig 10 we report a similar visualization to Fig 9, this time using the multi-resolution approach displaying four instances of superpixel segmentation.

**Fig 11:**
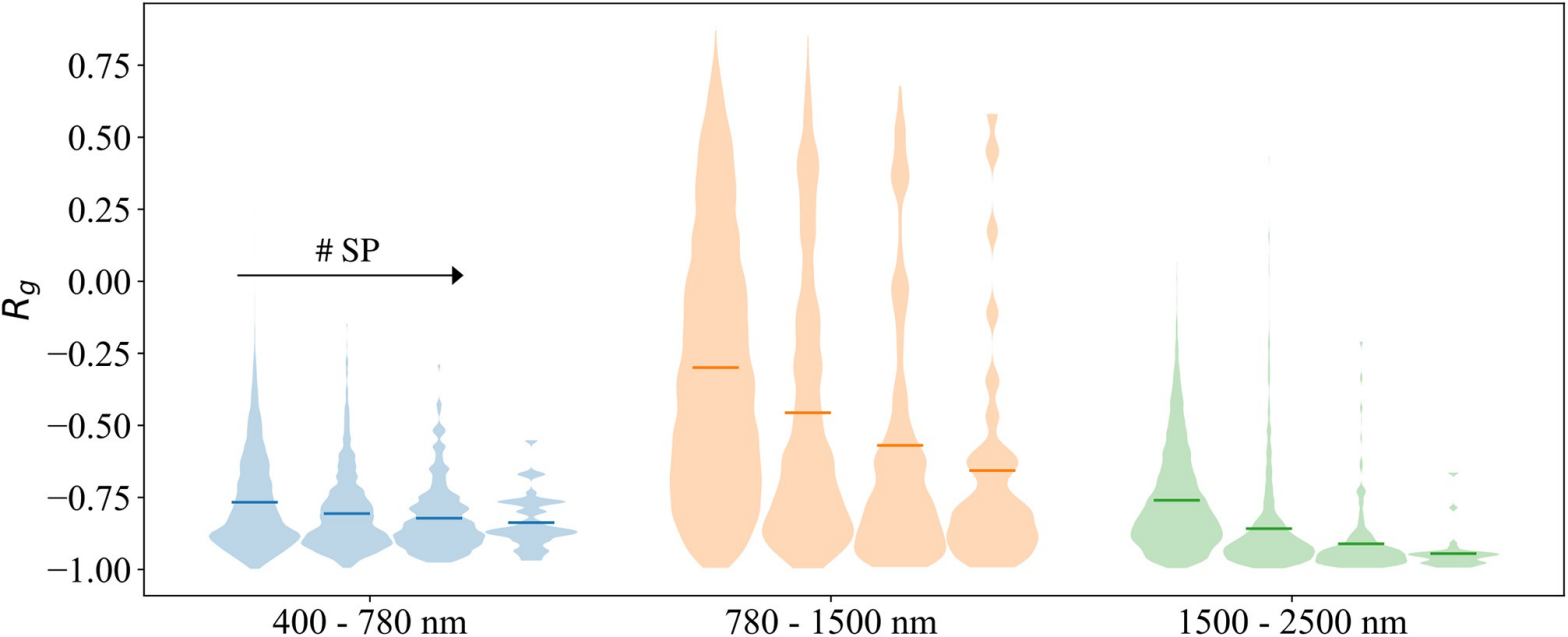
Global correlation distributions from the scenes of Fig 10. The horizontal lines represent the mean of each scene, with values ranging from -0.77 to -0.84 for the different resolutions in the visible range (400-780 nm), -0.30 to -0.66 in the NIR range (780-1500 nm), and -0.76 to -0.95 in the SWIR range (1500-2500 nm).

As support to Fig 10, we illustrate the distribution of correlation values for each scene in Fig 11.

By further studying the original hypothesis formulated by Le Hors, it became soon evident how they are based on assumptions that in real-case scenarios are seldom respected. In the context of imaging a real painting, unlike in the case in which smooth mockups are considered, it is necessary to consider the further level of complexity provided by the local surface topology around a pixel.

Let us consider for instance the first column of Fig 10. Upon visual inspection of the painting, it is possible to observe that the areas showing the strongest negative correlation are those in which the surface is relatively smoother. As a possible explanation for this, we argue that when the complexity of the topology is increased, surface scattering plays a more prominent role, thus keeping both reflectance and degree of linear polarization at high levels and correlated at times.

As we shift towards coarser scale representations, the global correlation becomes ever more negative. Indeed, grouping pixels in a local neighborhood is equivalent to reducing the complexity of the surface topology. However, the spatial resolution in this context can represent a bottleneck for the evaluation of polarimetric properties. A too-fine pixel representation can result in noisy data, whereas a too-coarse grouping can flatten the surface and lead to misinterpretations.

The recorded degree of linear polarization is however dependent on the relative angle between the incident light and the surface normal. We argue that a change in illumination direction, which would fade away from imaging standards in Cultural Heritage digitization, will produce a different distribution of specular reflections, but would not change the macro-observation formulated so far, as the surface of the painting is too complex and the resulting distribution of incidence angles would not be too dissimilar. At the same time, it is plausible that this assumption does not hold for angles that approximate the modalities of image capturing with raking light.

**Fig 12:**
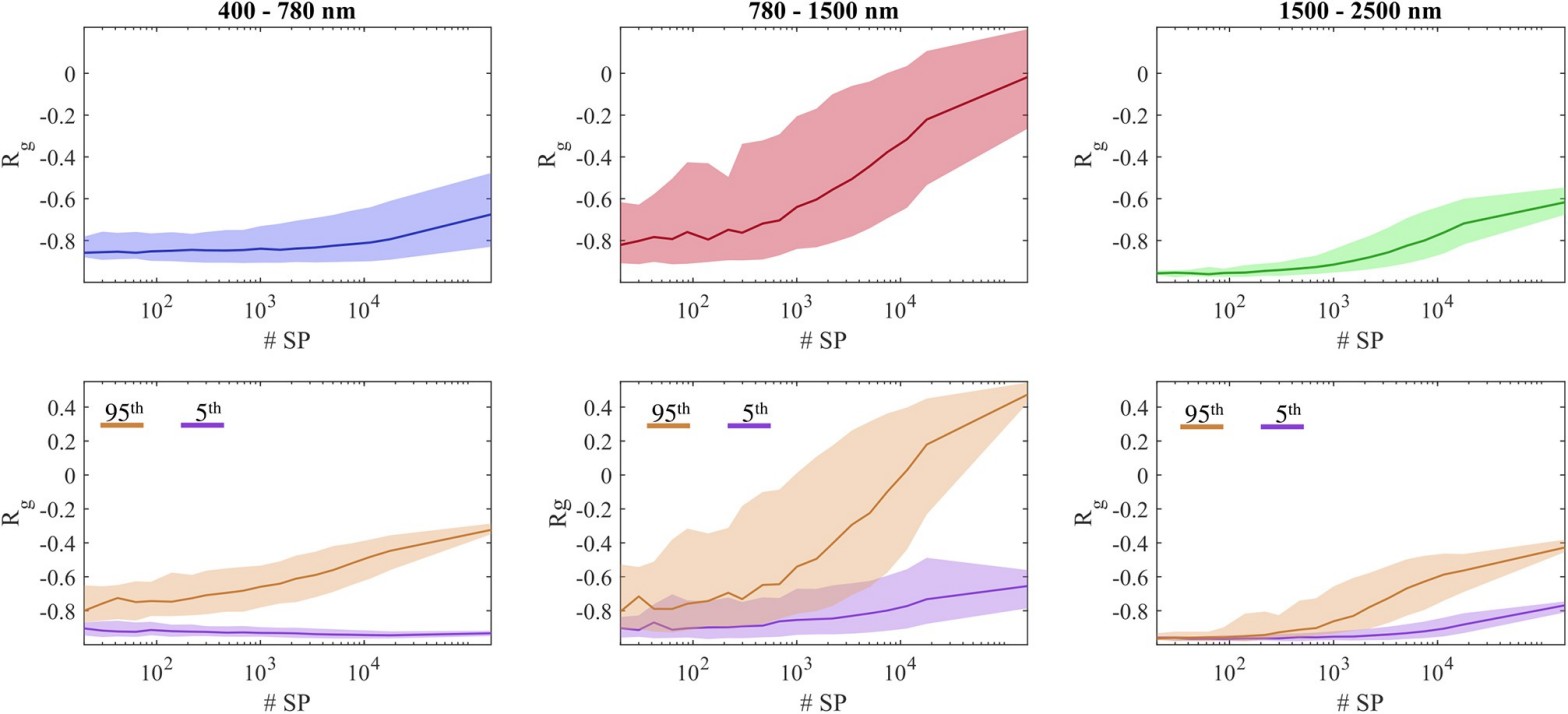
Global correlation as a function of scale expressed in terms of the number of superpixels in which the scene is segmented. Top row: median correlation value of all superpixels and relative interquartile range in the shaded areas. Bottom row: median correlation in the groups of pixels that result in the 5^th^ and 95^th^ percentiles and corresponding interquartile range in the shaded areas.

The top row of Fig 12 reports the behavior of the global correlation as a function of the number of deployed superpixels. In this case, *R*_*g*_ is the average of all superpixels (or pixels, in the case of the original image) in the scene. In the bottom row of Fig 12 two groups of pixels are identified in the correlation maps displayed in Fig 9: those with a *R*_*g*_ value lower than the 5^th^ percentile, and those with a *R*_*g*_ greater than the 95^th^ percentile. The values of these groups of pixels are then tracked through the various resolution steps and plotted. In both rows, the shaded areas illustrate the interquartile range.

**Fig 13:**
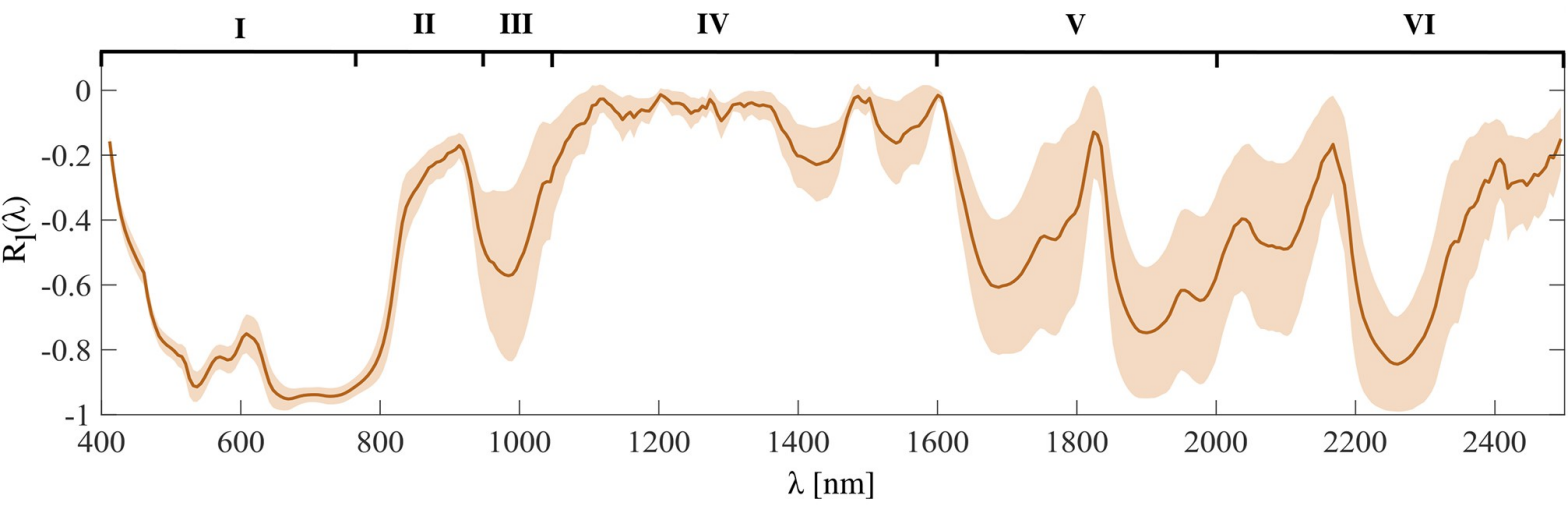
Mean local correlation *R*_*l*_(λ) between *S*_0_ and *ρ* as a function of wavelength for all scales. The local correlation is computed at each wavelength in a window of 19 data points, which correspond to approximately 104 nm. The shaded area corresponds to the computed standard deviation. The subdivision in Sectors is arbitrarily performed according to the shape properties of the curve except for Sectors V and VI, where the division is dictated by the fact that the nominal operating range of the analysis polarizer stops at 2000 nm.

Fig 13 illustrates the local spectral correlation *R*_*l*_ as a function of the wavelength. In order to compute this measure it is important to harmonize the spectral sampling, which is different in the VNIR and SWIR ranges. The decision is to interpolate the sampling in the VNIR range to the spectral resolution of the SWIR range (5.45 nm). The width of the window is set to 19 bands (approximately 104 nm), but it can be selected arbitrarily, keeping in mind that a window too small will output a noisy curve, while as the window size is increased, the global correlation *R*_*g*_ is approached. The curve reports the mean value of *R*_*l*_(λ) computed for all resolution steps, for the whole image. Similarly to the previous plots, the shaded area represents the interquartile range.

The plot of Fig 13 has been segmented into six relevant spectral intervals (numbered I, II, III, IV, V, VI) for ease of discussion.

Sector I, representative of the visible range from 400 nm to 780 nm, shows a mild negative correlation followed by a strong negative correlation until its end. It is likely that the first spectral bands are affected by sensor noise and higher polarization sensitivity, as observed by Lenhard *et al.* for similar devices [40].

The first bands of the infrared (Sector II, 780 nm to 950 nm) display a progressive decorrelation followed by a dip (Sector III, 950 nm—1050 nm), and then complete decorrelation in Sector IV between 1050 nm and 1600 nm. We argue that the dip observed in Sector III could be an artificial result produced either by the change of analysis polarizer in front of the spectral sensor, by the splicing correction, or both.

The deeper Sectors located in the infrared (V from 1600 nm to 2000 nm, and VI from 2000 nm to 2500 nm) display a varying correlation ranging from 0 to -1. The shape of the curve in these intervals could be related to the more spiky nature of infrared reflectance spectra, which have typically narrow absorption bands. It is noteworthy that the analysis polarizer used in front of the SWIR camera has defined specifics up to 2000 nm (Fig 3, hence the division in Sectors V and VI), and its behavior is unknown after this critical wavelength.

## 5 Conclusion

We designed an efficient spectro-polarimetric system for the acquisition of polarimetric images in combination with reflectance imaging spectroscopy in the VNIR and SWIR. This work is placed in a broader context that aims at achieving deeper material analysis in the context of Cultural Heritage once a Mueller imaging framework is implemented. The main challenge to achieving low-noise Mueller imaging in a wide spectral range resides in the development of broadband circular polarizers (as most use λ-selective phase delayers) to be deployed in the Polarization State Generator. At the present stage, the main goal was to implement a spectral linear Stokes imaging framework with low noise, as well as to study the correlation between (pseudo) reflectance (the first element of the Stokes vector *S*_0_) and degree of linear polarization in a mockup oil painting.

We observe that the correlation between the degree of linear polarization and the amount of reflected radiation is in general negative in the visible range, while a decorrelation is observed in the first part of the infrared range between 780 nm and 1500 nm. Contrarily to the trend, the second part of the infrared (between 1500 nm and 2500 nm) displays a strengthening of the negative correlation. We ascribe these differences to the changes in relative quantities of specular components, surface scattering, and volume scattering, but a fully dedicated study can only be beneficial to explain the observations we made. Moreover, we observe that the correlation depends on the surface topology, as complex local neighborhoods tend to display weaker correlations. This is corroborated by a multi-resolution analysis in which coarser representations of the scene show stronger correlations, due to the fact that the surface is artificially flattened.

The limitations of our system are related to the usage of two separate imaging sensors that need to be spliced around 1000 nm, and to the possible inaccuracy of the analysis polarizer after 2000 nm. Thus, our observations in these intervals need to be consolidated.

Paintings and historical artifacts typically feature complex surface geometries that represent challenging instances of light-matter interactions. On top of that, experimental setups can adopt various illumination geometries that can make the characterization of surfaces even more intricate. Our study was limited to studying the relationship between reflected radiation and its degree of linear polarization, but many more effects take place. A full phenomenological study can then include factors such as dependencies of acquisition geometries (illumination and observation angles), and characterization of the angle of linear polarization.

Furthermore, we argue that the proposed observations should be verified for other dielectric materials and it is likely that they will not be valid for conductive surfaces.

A future line of work can include the spectro-polarimetric characterization of typical materials used in art paintings for the construction of features to be exploited in operations of spectral unmixing and pigment mapping.
